# The Role of Sialyl-Tn in Cancer

**DOI:** 10.3390/ijms17030275

**Published:** 2016-02-24

**Authors:** Jennifer Munkley

**Affiliations:** Institute of Genetic Medicine, Newcastle University, Newcastle-upon-Tyne NE1 3BZ, UK; jennifer.munkley@ncl.ac.uk; Tel.: +44-(0)191-241-8616

**Keywords:** sialyl-Tn, cancer, *O*-glycans, ST6GalNAc1, *COSMC*, glycosylation

## Abstract

Activation of an aberrant glycosylation pathway in cancer cells can lead to expression of the onco-foetal sialyl-Tn (sTn) antigen. STn is a truncated *O*-glycan containing a sialic acid α-2,6 linked to GalNAc α-*O*-Ser/Thr and is associated with an adverse outcome and poor prognosis in cancer patients. The biosynthesis of the sTn antigen has been linked to the expression of the sialytransferase ST6GalNAc1, and also to mutations in and loss of heterozygosity of the *COSMC* gene. sTn neo- or over-expression occurs in many types of epithelial cancer including gastric, colon, breast, lung, oesophageal, prostate and endometrial cancer. sTn is believed to be carried by a variety of glycoproteins and may influence protein function and be involved in tumour development. This review discusses how the role of sTn in cancer development and tumour cell invasiveness might be organ specific and occur through different mechanisms depending on each cancer type or subtype. As the sTn-antigen is expressed early in carcinogenesis targeting sTn in cancer may enable the targeting of tumours from the earliest stage.

## 1. Introduction

Aberrant Glycosylation can play an important role in key fundamental processes occurring in cancer and changes in *O*-glycosylation are frequently observed [1]. *O*-glycosylation of proteins is a diverse and abundant form of post-translational modification that occurs in the Golgi apparatus and depends on the sequential action of several glycosylation enzymes. The biosynthesis of *O*-glycans is initiated by a family of up to 20 GalNAc-transferases that transfer *N*-Acetylgalactosamine (GalNAc) to serine threonine residues on proteins to produce the Tn antigen. The Tn antigen is then further branched and capped in subsequent processing steps by a large number of different glycosyltransferases. In normal cells, *O*-glycosylation proceeds to mature elongated and branched *O*-glycans, which are frequently modified with sialic acid. An increase in global sialylation has been closely associated with cancer and can play a fundamental role in cell adhesion, cellular recognition and cell signalling [1,2].

The pattern of glycans expressed in a cell depends on the glycosyltransferases expressed, their substrate specificity, and localisation. In cancer cells the processing of *O*-glycans to mature branched structures is often disrupted, and they express only early biosynthetic intermediates [3,4]. The sialyl-Tn antigen (Neu5Acα2-6GalNAcα-*O*-Ser/Thr), known as sTn, is a truncated *O*-glycan containing a sialic acid α-2,6 linked to GalNAc α-*O*-Serine/Threonine (Ser/Thr). sTn is expressed by more than 80% of human carcinomas and is linked to poor prognosis in cancer patients. The sTn antigen was first discovered as a cancer marker >30 years ago. Early studies identified the sTn as a marker for diagnosis and prognosis in cancer, and later work focused on using sTn as a potential therapeutic target. sTn neo- or over-expression has been described in many types of epithelial cancer including: gastric [5], colon [6], breast [7,8], lung [9], oesophageal [10], pancreatic [11], prostate [12,13], bladder [14], and endometrial cancer [15]. Studies suggest that sTn expression has primarily been detected at the apical or luminal surface of tissue and tends to occur in early carcinogenesis [16].

## 2. Synthesis of the Sialyl-Tn (sTn) Antigen

**Figure 1:**
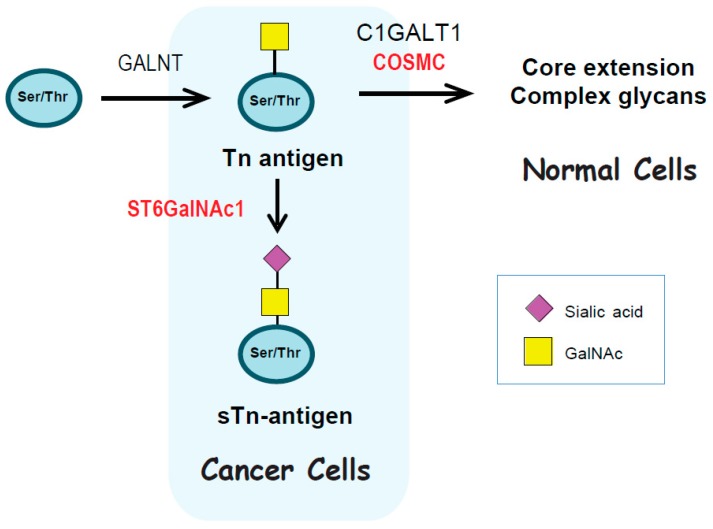
Biosynthesis of the Sialyl-Tn (sTn) antigen in cancer cells.

The expression of truncated *O*-glycans in cancer has been proposed to result from altered expression of glycosyltransferases [17], hypermethylation or mutations in *COSMC* (core 1 β3-Gal-T-specific molecular chaperone) [18,19], mis-localisation of GalNAc-transferases from the Golgi to the endoplasmic reticulum (ER) [20], and fluctuations in pH levels [21]. Specifically, biosynthesis of the sTn antigen has been linked to the expression of the sialytransferase ST6GalNAc1, and to mutations in and loss of heterozygosity of the *COSMC* gene (Figure 1). The biosynthesis of sTn can be mediated *in vitro* by the specific sialyltransferases ST6GalNAc1 and ST6GalNAc2 [22,23]. However, in a cellular context only ST6GalNAc1 is able to synthesise sTn [17,22], and over-expression of ST6GalNAc1 in gastric, breast and prostate cell lines has been shown to induce expression of sTn, indicating a crucial role for this enzyme in sTn biosynthesis [17,24,25,26].

The enzyme C1Ga1T1 catalyses the common first elongation step of the Tn antigen to produce the core 1 *O*-glycan structure (T antigen) and requires the COSMC chaperone protein for correct folding and activity [18,27,28,29]. Mutations in and loss of heterozygosity of the *COSMC* gene have been identified in sTn positive colon and melanoma cell lines, and also in sTn positive cervical cancer tissue [18]. Although these events are thought to be rare in breast and colon cancer [30], up to 40% of cases of pancreatic cancer have been found to have hypermethylation of the *COSMC* gene, and a corresponding increase in truncated *O*-glycans [19]. Increased expression of sTn in cancer may also be enhanced by increased synthesis of the *O*-GalNAc precursor and decreased competition due to reduced core-synthesis.

## 3. sTn Is an Onco-Foetal Antigen

Monoclonal antibodies specific to sTn have been used to show that overall expression of sTn is rare in normal compared to cancer tissues, leading to sTn being described as an onco-foetal antigen [16]. However, while there are some studies describing sTn detection in foetal organs and amniotic fluids [7,31,32,33], a role for sTn in embryonic development remains to be identified. In adult healthy tissues, low sTn expression has been detected in the salivary gland, oesophagus and stomach, and appears to be restricted to the upper digestive tract [7,34,35]. Expression of sTn has also been linked to inflammatory diseases of the digestive tract and may be regulated by ƴ-interferon inflammatory signals [36,37,38]. sTn is believed to be carried by a number of glycoproteins which are differentially expressed in different cell types [16]. In mucinous cells, sTn is carried by glycoproteins such as mucins which can be either membrane bound and secreted [39,40]. Squamous cells mainly have intensive staining in the cytoplasm and some staining on the cell membrane [10,41]. MUC1 (mucin 1, cell surface associated), CD44 (CD44 molecule, Indian blood group), integrin β1 and osteopontin proteins have been identified as sTn carrier proteins [24,42,43,44], all of which have roles in cell adhesion, migration or chemotaxis. It is thought that the addition of sTn to these glycoproteins may influence protein function and play a role in specific mechanisms involved in tumour development [45].

## 4. sTn as a Prognostic Biomarker

Detection of serum tumour markers is a non-invasive, simple method for diagnosis and post-surgery analysis. The sTn antigen enters serum through *O*-glycoprotein shedding or secretion from tumours into the bloodstream. This requires a large amount of tumour mass, which is more likely found in advanced cancers and is therefore associated with poor prognosis. A high level of sTn (>38 U/mL) has been detected in the sera of patients with gastric, colorectal, pancreatic, cervical, endometrial and ovarian cancers [15,46,47,48,49,50,51,52,53], and is associated with tumour size and metastasis [46,47,54]. In prostate cancer, sTn is detected in up to half of all high grade tumours [12,13], and sTn-MUC1 has been correlated with survival outcome and higher serum Prostate Specific Antigen (PSA) levels [55]. A recent study has also linked elevated serum sTn levels to histological grade and lymph node metastasis in endometrial cancer [15].

While sTn has been associated with decreased overall survival of patients, this may depend on the type of cancer studied [16]. For example, in lung cancer, no link to survival was found [56], and in prostate cancer the STGalNAc1 enzyme was found to be up-regulated in primary prostate cancer tissue but significantly down-regulated in metastatic lesions [25]. Studies in these cancer types have been limited, but is hoped that additional investigations will help further determine the prognostic value of sTn in different cancer types.

## 5. The Role of sTn in Cancer Progression

Truncated *O*-glycans occur on the majority of epithelial cancers and in many pre-malignant lesions [11,57,58]. A recent study by Radhakrishnan *et al.* showed that immature truncated *O*-glycans can directly induce oncogenic features in cancer cells, including increased proliferation, loss of tissue architecture, disruption of basement membrane adhesion and invasive growth [19]. In breast and gastric cancer cell lines, sTn up-regulation is linked to increased tumour growth and metastases in *in vivo* models [24,42]. Although induction of sTn inhibited the formation of stable tumour masses in prostate cancer, potentially promoting cancer cell dissemination, there was no effect upon metastasis [25]. Consistent with this, sTn positive ovarian cancer cells are more frequently observed at the invasive front of tumours but less often in metastatic lesions [59,60], and breast ductal invasive carcinomas have lower levels of sTn than primary ductal carcinomas [61]. Studies have suggested that sTn can play a role in protecting metastatic cells in the blood stream from degradation by the immune system [62]. Alterations in glycoprotein composition on the cell surface can induce or prevent the recognition by lectin molecules such as selectins, siglecs and galectins, which can play a role in cell–cell and cell–matrix interactions, and may influence cancer progression [63]. As the biosynthesis of sTn gives rise to negative charges, sTn expression is implicated in the interaction of cells with their surrounding environment [64]. It is likely that the sTn-antigen facilitates the release of individual cells from the primary tumour mass by reducing cell–cell aggregation, for example by disrupting the interaction of galectins with terminal galactose residues [65,66]. Thus, in some cases, sTn may enhance the dissemination of cancer cells, promoting primary tumour transition, but may not improve the settlement of metastatic cells at secondary sites [25]. Cells expressing sTn may have an improved ability to migrate and invade underlying tissue and eventually reach blood or lymph vessels [67]. As specific adhesive properties are needed for successful extravasation of metastatic cells and invasion of target tissue, one possibility is that sTn can sometimes play a transient role in cancer progression [25].

Targeting sTn carrier proteins is a potential cancer therapeutic option. It remains open whether sTn might be carried on cancer stem cell marker proteins but as these proteins can be modified with *O*-glycans this seems a likely possibility [68]. The expression of sTn is generally reported to be heterogeneous within tumours [39,69,70,71,72,73] and could be regulated in the tumour via the expression of carrier proteins. As sTn can be carried by different glycoproteins in different compartments, its role in cancer progression may be different in different tumour types, and the effect of sTn expression on tumour cell invasiveness might be organ specific. Together these findings suggest that the biological function of sTn in promoting cancer development may occur through different mechanisms depending on each cancer type or subtype. Future studies using animal models expressing sTn in multiple cancer types and cell backgrounds will help further elucidate the role in sTn in cancer cell invasion and metastasis in different cancers. 

Immunotherapy, or using the patient’s own immune system to target cancer cells, is an attractive approach to treat cancer, and the side-effects are mild compared with traditional therapies. Anti-tumour antibodies could delay tumour growth by antibody dependent cytotoxicity and inhibition of function. Since healthy adult epithelial cells do not normally expose the sTn antigen to the immune system and bloodstream, the use of sTn as an immunisation antigen is an attractive option [16,74,75]. Clinical studies have demonstrated that immunisation with anti-sTn vaccines can induce production of sTn specific IgGs [76,77,78], and in murine models sTn vaccines have been found to induce antibody mediated tumour protection [44]. Additionally, in early stage breast cancer, the detection of antibodies against MUC1-sTn is associated with increased time to metastasis suggesting a protective role of anti-sTn antibodies [79]. sTn antibodies have been detected in healthy females who did not go on to develop cancer 25–30 years after blood sampling, suggesting a possible functional role or these antibodies in suppressing tumour development [79].

The Theratope anti-sTn vaccine produces an anti-sTn immune response and is well tolerated by patients with little toxicity [80,81,82]. In phase II clinical trials for patients with metastatic breast cancer there was a significant improvement in survival by 12 months [80,81,82,83,84]. These promising results led to a randomised, double blind phase III trial involving over 1000 women with metastatic breast cancer, but unfortunately in this trial Theratope failed to demonstrate improved patient survival or time to disease progression [76]. One possible explanation for the failure of the Theratope phase III clinical trial is that the patients were not pre-evaluated for sTn expression, meaning that heterogeneous sTn expression between patients could have masked any benefit of the vaccine [16]. A concurrent study showed that Theratope did increase survival in a pre-stratified subset of patients who were receiving hormonal therapy [85]. The reasons why anti-sTn immunisation seems to only be effective in a subset of patients remain unclear but the overall results point to a relative efficiency and safety of the Theratope vaccine.

In recent years, there has been rapid progress in antibody production methodology, including the immortalisation of human B cells, phage display technology and high throughput screening, transgenic mice and more recently molecular-engineered antibodies [86]. It is hoped these new technologies may have the potential to develop novel antibodies against sTn for use in cancer treatment [86]. An immunotherapy approach targeting sTn carrier proteins may offer the possibility to target more specific mechanisms in tumour development. For example, vaccines targeting MUC1-sTn have been evaluated in clinical trials (Bradbury and Shepherd, 2008, Beatson *et al.*, 2010). MUC1 is expressed on the surface of epithelial cells where it is involved in cell adhesion and signalling (Hollingsworth and Swanson, 2004), and can be aberrantly glycosylated with sTn (Burdick *et al.*, 1997). It may also be possible to create cell therapy via training dendritic cells (DCs) or T cells with sTn or sTn carrier proteins as a tool for cancer therapy. For anti-sTn therapy to advance further it will be vital to fully understand the role that the sTn antigen plays in the function of sTn expressing proteins and in overall tumour progression. Once this is achieved, it may also be possible to combine sTn with other cancer associated antigens in order to trigger multi-antigenic responses in patients to create new platforms for delivery of anti-tumour therapeutics based on the patient’s personal tumour profile (Niederhafner *et al.*, 2008, Slovin, 2007). As the sTn-antigen is expressed early in carcinogenesis in all epithelial cancers investigated, targeting sTn in cancer may enable the targeting of tumours from the earliest stage.

## 6. Future Perspectives

Many proteins with central roles in cell–cell adhesion, differentiation and development are *O*-glycosylated and known to have roles in cancer [19,87]. Induction of sTn in cancer could potentially produce global effects on *O*-glycosylation of proteins, and simultaneously affect multiple systems to promote tumour formation and growth, meaning that deciphering the molecular mechanisms underlying the role of sTn in cancer will not be straightforward. Site specific modification of proteins with *O*-glycans can have profound effects on protein functions such as pro-protein processing, modulation of ligand binding properties of receptors and regulation of cell signalling [88,89,90]. Future studies determining the specific effects of sTn on specific glycoproteins will help further dissect the role of sTn in cancer.
